# What can the Thatcher illusion tell us about face processing in the brain? Commentary on Psalta, Young, Thompson, and Andrews (2014)

**DOI:** 10.3389/fnhum.2014.00289

**Published:** 2014-05-08

**Authors:** Constantin Rezlescu, Tirta Susilo

**Affiliations:** ^1^Department of Psychology, Harvard UniversityCambridge, MA, USA; ^2^Department of Psychological and Brain Sciences, Dartmouth CollegeHanover, NH, USA

**Keywords:** face processing, face expressions, thatcher illusion, superior temporal sulcus, fusiform face area

Three decades ago Thompson (1980) discovered what is now a classic visual demonstration in psychology: that inverting the eyes and mouth of Margaret Thatcher's face passes largely unnoticed when the face is presented upside-down, even though the face appears grotesque in the upright orientation. Recently, Psalta et al. (2014) used fMRI to identify the neural basis of the Thatcher illusion and found that the face-selective superior temporal sulcus (fSTS), but not the fusiform face area (FFA) or the occipital face area (OFA), tracked the grotesque appearance of upright but not inverted Thatcherized faces. Referring to fSTS, the authors conclude: “*Our results demonstrate clear evidence for orientation-dependent sensitivity to changes in facial expression in a key component of the neural network underlying face perception*.” While we agree that fSTS is involved in processing face expression (Haxby and Gobbini, 2011), based on conceptual and statistical issues we argue that the conclusion of Psalta et al. (2014) is not supported by the data.

## Conceptual issues

To claim that the study provides evidence for orientation-sensitive processing of face expression in fSTS, at least one of the following has to be true:

1. The Thatcher effect measured in the current study concerns face expression and nothing else
2. fSTS is involved in processing face expression and nothing else

Several considerations cast doubt on (1). First, there are no theoretical or empirical grounds for an exclusive link between the Thatcher illusion and face expressions. For example, Bartlett and Searcy (1993) showed that the grotesqueness of the Thatcher illusion is caused not by the expression of the face but rather by the disruption of the normal face configuration, which led them to argue: “*The Thatcher illusion cannot be explained as an effect of inversion on the encoding of expression*.” (p. 311). Moreover, it is not clear whether the grotesque appearance of the Thatcher illusion is best described as an expression of the face or as a feeling of the viewer invoked by the bizarreness of the image. Unlike stimuli commonly used to study expression processing such as happy and angry faces, Thatcherized faces do not convey the mental states of the person depicted. This consideration suggests that Thatcherized faces are at best unusual stimuli for investigating expression processing as typically conceived in the literature (Calder, 2011).

Second, Psalta et al. (2014) did not have a control condition that deconfounds face expression from other factors likely involved in the Thatcher illusion. Without this control, it remains possible that the fSTS effect is driven by the bizarreness of the stimulus or other confounds rather than the expression of the face *per se*. Indeed, the result could have been taken as evidence that fSTS shows orientation sensitivity to face configuration, face distortion, or face bizarreness in the upright orientation.

Statement (2) is also not true because many studies have shown that fSTS is a functionally heterogeneous region implicated in processing many face aspects other than expression. For example, fSTS is involved in processing of eye gaze (Engell and Haxby, 2007), gaze irrespective of head view (Carlin et al., 2011), head turns (Carlin et al., 2012), dynamic but non-expressive faces (Pitcher et al., 2011), and face distinctiveness (Mattavelli et al., 2012). The involvement of fSTS in processing face distinctiveness is particularly relevant, because it offers an alternative interpretation of the results, namely that fSTS shows orientation sensitivity to face distortion that appears distinctive, in this case grotesque.

## Statistical issues

The results of Psalta et al. (2014) are also difficult to interpret because of two statistical concerns. First is the lack of correction for multiple comparisons despite the authors' tests for an interaction in three face-selective regions: “*Our reasoning was that any region that contributes to the perception of the Thatcher illusion should show a greater response … Moreover, this difference in response should be evident for upright but not inverted faces*.” Following this prediction they should have not used alpha of 0.05 but rather [0.05/3] = 0.017 (Bonferroni correction). When this correction is applied, the *p*-value for the key interaction in fSTS, *F*_(2, 46)_ = 3.03, *p* = 0.058 (not < 0.05 as incorrectly reported) is clearly not significant.

In addition, it is unclear whether the authors had an exclusive apriori interest on the interaction between face condition and orientation. If not, then a further correction for the two main effects and an interaction measured by each of the three Two-Way ANOVAs would have been necessary. On this view, the corrected alpha should have been [0.05/9] = 0.006, under which none of the statistical tests are significant.

A second issue is the fallacy of inferring that the difference between significance and non-significance is automatically significant (Gelman and Stern, 2006; Nieuwenhuis et al., 2011). Psalta et al. (2014) ran three separate Two-Way ANOVAs (condition × orientation), one for each face-selective region. A significant interaction in fSTS but not in FFA or OFA was summarized: “*the selectivity of the response in the STS can be seen by contrasting it with the responses of other face-selective regions*.” But this contrast may not be significant: the authors need to test whether the difference between significance in fSTS and non-significance in FFA and OFA is actually significant—a test that would have been performed had they began their analysis with a Three-Way ANOVA (region × condition × orientation).

Finally, these shortcomings related to null hypothesis testing could have been compensated by presentation of effect sizes and more informative figures (Wilcox, 2006; Allen et al., 2012). This is essential because *p* values around the significance threshold are notoriously difficult to interpret (Wetzels et al., 2011).

The Thatcher illusion is an intriguing visual phenomenon that can reveal deep insights about face processing in the brain. But Psalta et al. (2014)'s conclusion that fSTS demonstrates orientation sensitivity to changes in face expression does not follow from their data.

### Conflict of interest statement

The authors declare that the research was conducted in the absence of any commercial or financial relationships that could be construed as a potential conflict of interest.
